# A digital reconstruction of the 1630–1631 large plague outbreak in Venice

**DOI:** 10.1038/s41598-020-74775-6

**Published:** 2020-10-20

**Authors:** Gianrocco Lazzari, Giovanni Colavizza, Fabio Bortoluzzi, Davide Drago, Andrea Erboso, Francesca Zugno, Frédéric Kaplan, Marcel Salathé

**Affiliations:** 1 Digital Epidemiology Laboratory, School of Life Sciences, École Polytechnique Fédérale de Lausanne (EPFL), Lausanne, Switzerland; 2 Institute for Logic, Language and Computation (ILLC), University of Amsterdam, Amsterdam, The Netherlands; 3 Digital Humanities Laboratory, College of Humanities, École Polytechnique Fédérale de Lausanne (EPFL), Lausanne, Switzerland

**Keywords:** Bacterial infection, Computational models

## Abstract

The plague, an infectious disease caused by the bacterium *Yersinia pestis*, is widely considered to be responsible for the most devastating and deadly pandemics in human history. Starting with the infamous *Black Death*, plague outbreaks are estimated to have killed around 100 million people over multiple centuries, with local mortality rates as high as 60%. However, detailed pictures of the disease dynamics of these outbreaks centuries ago remain scarce, mainly due to the lack of high-quality historical data in digital form. Here, we present an analysis of the 1630–1631 plague outbreak in the city of Venice, using newly collected daily death records. We identify the presence of a two-peak pattern, for which we present two possible explanations based on computational models of disease dynamics. Systematically digitized historical records like the ones presented here promise to enrich our understanding of historical phenomena of enduring importance. This work contributes to the recently renewed interdisciplinary foray into the epidemiological and societal impact of pre-modern epidemics.

## Introduction

“*Sia laudato il signor Iddio non ci sono stati morti*.”

Bless the Lord, there have been no deaths [today].

December 24th 1630, in *Sant’Eufemia*, Venice.

Disease outbreaks of the plague in the past centuries have been so devastating throughout Eurasia that the very term *plague* has become synonymous with a devastating epidemic. By killing a substantial proportion of the human population, which took multiple generations to recover, plague pandemics have had enormous impacts on the development of Eurasia. Correspondingly, historical questions, such as the role of institutions and the socioeconomic impact of plague outbreaks^1^, as well as epidemiological questions, such as the causes, nature and interactions of vectors^2–5^, seasonality and climatic patterns^6,7^ and even the distinction between plague and the Black Death^8^, are still being investigated. While previous studies have highlighted some common traits to plague epidemics^9^, such as the high impact on densely-inhabited cities acting as hotspots^10,11^, the importance of human-to-human transmission^12^ and the effect of the plague on different sexes^13^, little is known about local outbreaks, due to the lack of detailed historical data.

We analyze high-quality data from death records created during the 1630–1631 plague epidemic in Venice, whose initial investigation is limited and by now dated^14^. This epidemic was part of the so-called “Second Pandemic”, which started with the Black Death and lasted until the early 19th century. Originating in northern Europe (modern France and the Rhineland) in 1623, this epidemic crossed the Alps approximately in 1629, in the case of the territories of the Republic of Venice likely carried by imperial armies on their way to Mantua. The cause of this specific outbreak in Venice has been linked to the bacterial species *Yersinia pestis*^15^, and with a set of surprising results, including an uneven and unexpected impact on different cohorts by sex and age, a high parallel increase of mortality due to a synchronous smallpox epidemic and a raise in public violence^16^.

**Figure 1:**
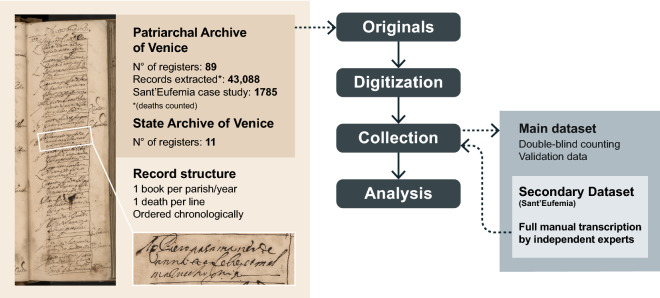
Illustration of the data collection workflow and datasets, including an example page from a death records book. The zoomed-in registration reads as follows: “*Messer Piero pasamaner de anni 40 febre et mal mazuccho giorni 5*”, which roughly translates to “Mister Piero passementerie’s weaver aged 40 fever and plague 5 days.” What is meant is that Mister Piero, a passementerie’s weaver forty of age (approximately), died of fever and plague after five days of sickness. This occurred on the 23rd of October, 1630 (as it can be read at the top of the page).

Venetian death records from this period, also referred to as *necrologies*, are organized by parish and contain the systematic registration of every death among the resident population. These necrologies, edited by the parson, were established by decree since 1504 and kept in the archives of the responsible magistracy^17^. While death records were commonplace in all Christendom since the late Middle ages, and are commonly used for demography studies including on the plague^1,18^, Venetian records were particularly detailed. In the Patriarchal Archives of Venice, 54 out of more than 70 existing parishes at the time still possess at least part of the registrations for the plague year (September 1630 to September 1631), while in the State Archive of Venice, the extant records for the plague year are few and scattered. Based on our assessments, these record series are overlapping and one (the former) constitutes the source for the other (the latter). We thus focus our efforts on the Patriarchal records. An example page from a necrology record is shown in Fig. 1. Necrology records were kept in tiny and oblong books, with entries grouped chronologically by day. Typically, the most recurring details given for every entry were: the name, profession, sex and age of the person, the cause of death, approximate length of illness and whether a doctor attended them or not. The main dataset we use in what follows contains the number of daily deaths per parish. Data were collected following the work-flow illustrated in Fig. 1; more details are given in the SI.

**Figure 2:**
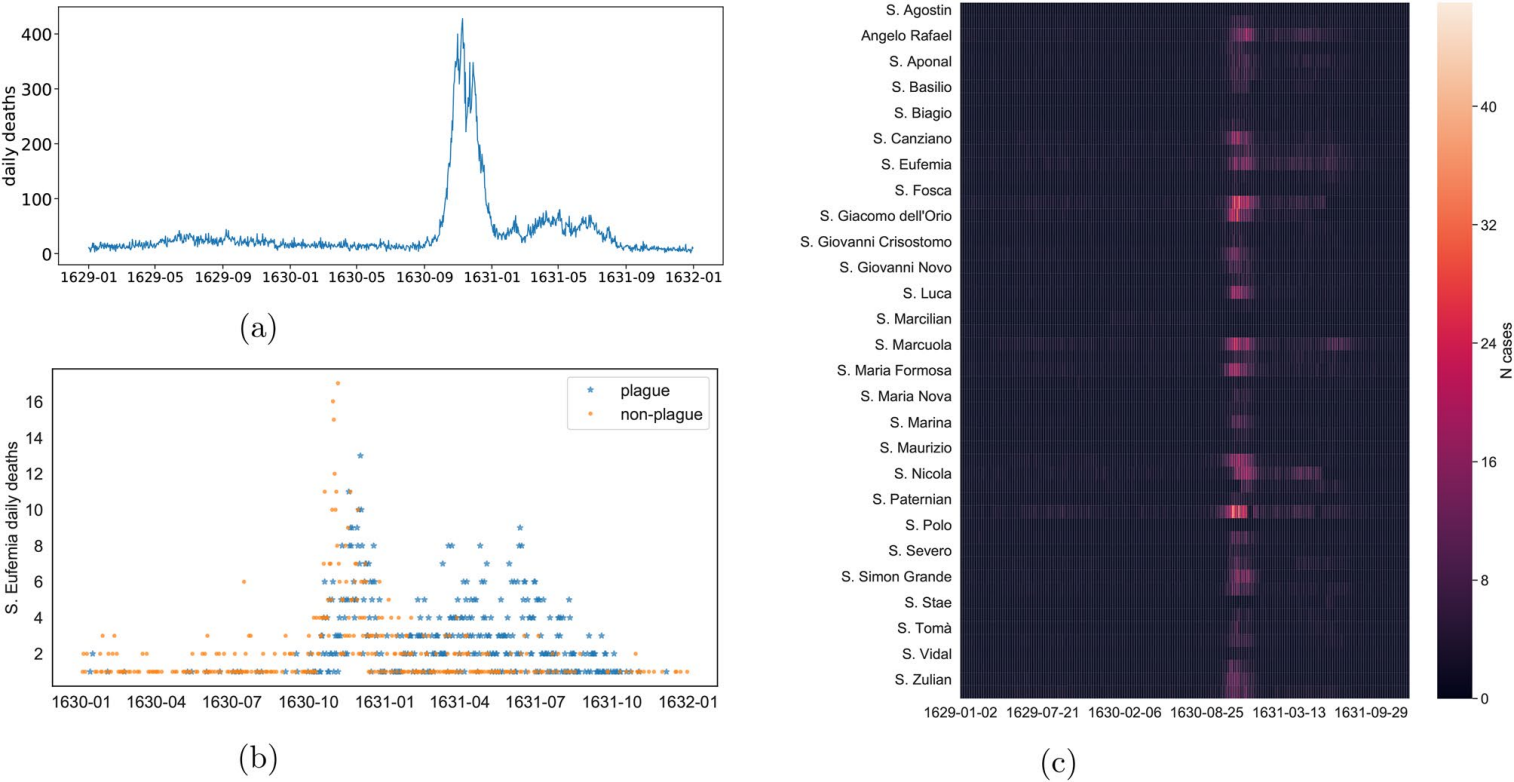
An overview of the full plague outbreak (main dataset): (**a**) Cumulative daily deaths for the whole recorded period (1095 days in total). A total number of 43,088 deaths were reported. One can clearly see the presence of a two-stage process, spanning until fall 1631. (**b**) Daily deaths recorded in the parish of *Sant’Eufemia*, almost surely due to plague (blue stars—$N_{plague} = 1007$) and possibly to other causes (orange circles—$N_{not \, plague} = 778$). Only days when someone died are considered. (**c**) A heatmap view of the dataset; for the sake of clarity, not all parishes names are plotted.

Our data aggregated over all parishes clearly shows the massive outbreak which took place between the September and December of 1630, as detailed in Fig. 2a. The death counts are staggering: 20,923 deaths between September and December 1630 alone, followed by 10,430 between January and August 1631. In total, 43,088 deaths were recorded over just three years. These numbers are in line with the 35% estimated mortality in northern Italy during the same epidemic outbreak^1^, and should be compared to an estimated average annual mortality between 3.7 and 2.7% (but 29.7% for newly-born infants) during the whole seventeenth century^19,20^. We stress that not all death records survived, therefore these numbers must be taken to represent a lower bound of the actual death toll. Historical demographic sources, even though uncertain^21^, report a population of 141,625 inhabitants for Venice in 1624 and of 102,243 in 1633, a reduction of 27.81%^19,20^.

The presence of a single peak of deaths is common in plague outbreaks within densely populated regions and cities^5,6^. Its presence in Venice indicates that the authorities’ best efforts to contain the epidemic—for example by gathering all sick people in public hospitals or in their houses^16^—simply failed. The city was too densely populated and connected to leave any margin for containment (for space distribution of deaths see fig. SI1). In fact, as it can be seen in Fig. 2c, the outbreak in 1630 swept through the parishes practically in sync, as no discernible space correlation is present. However, while the outbreak in 1630 is known, the subsequent 1631 long tail of high mortality has not been described in the literature before.

**Figure 3:**
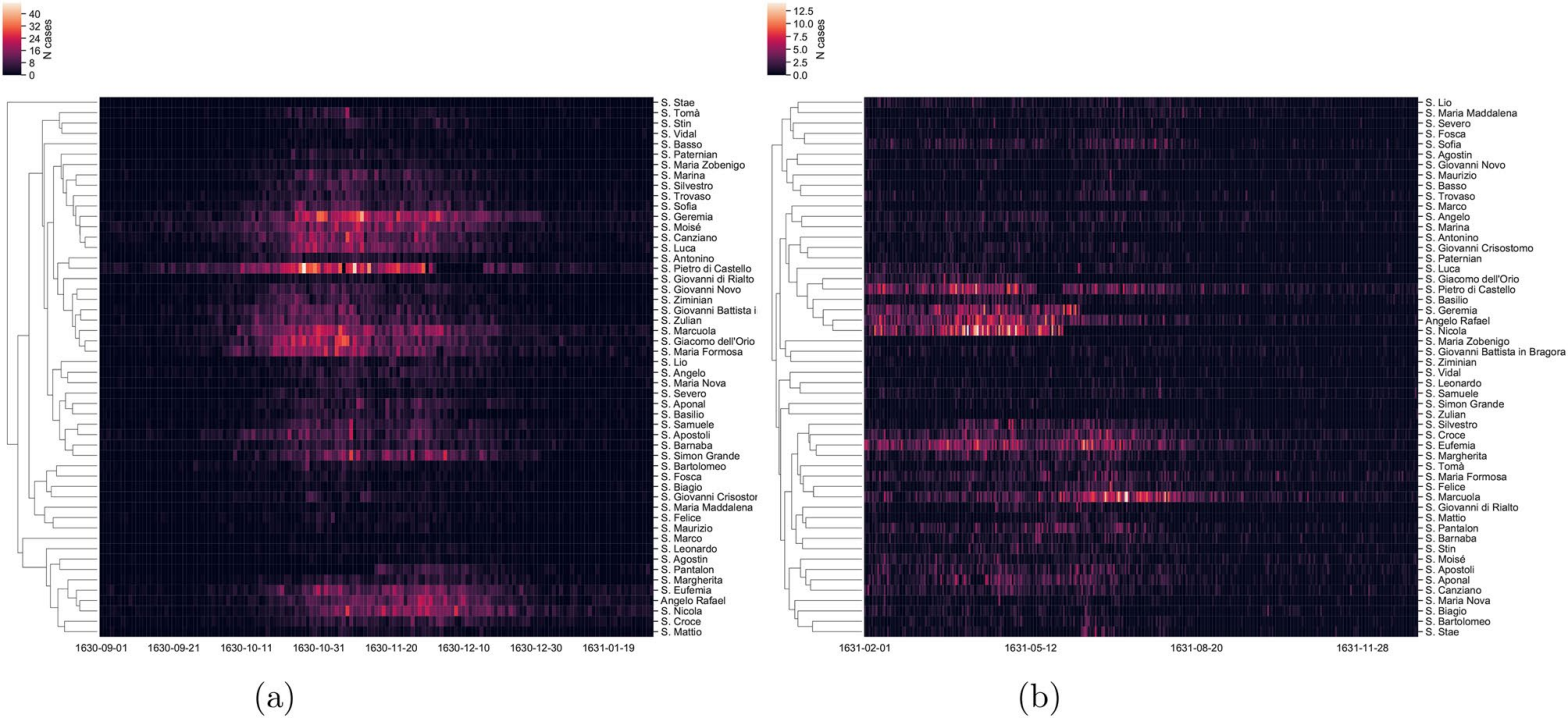
Hierarchical clustering of parishes zoomed on the main late-1630 peak (**a**) and on the 1631 outbreaks (**b**). The dendrograms on the light side of each sub-figure present the result of the clustering.

In order to gain a better understanding of the disease dynamics, we investigated another dataset taken from the records of a specific parish: *Sant’Eufemia*. This was a populous parish, with a significant amount of deaths in the 1631 tail and whose necrology records are well-preserved in their entirety. We transcribed all the information available in its necrologies, i.e. the name, sex and age at death of each person, together with the cause of death and the length of sickness. This transcription includes 1785 deaths registered between January 1630 and December 1631. The identification of deaths due to plague appears to be deceptively simple, as they were usually registered as fatalities caused by suspicious illness (“*mal sospetto*”), or with visible buboes. Nevertheless, previous studies have taken a more inclusive approach, considering also deaths not clearly caused by other factors as due to plague^16^. We take the more conservative approach in what follows—see Tables SI1, SI2 and SI3 for details on which causes of death were considered to be plague.

The statistics of the causes of death give us a first insight. In Figure SI2a we show the distribution of deaths grouped by cause and (conservatively) classified as related to the plague or not. One can see how the two distributions are skewed, meaning that a small fraction of causes (5%) contributes to a large fraction of deaths (63%). However, while the number of deaths clearly due to plague ($N_{plague} = 1007$) and possibly non-plague are similar ($N_{not \, plague} = 778$), only 56 out of 156 causes could be clearly attributed to plague, leaving more vagueness around the non-plague causes (in Figure SI2b the causes with more than 50 deaths are listed). This seems to suggest that our plague-death counts likely constitute a lower bound of the total number of deaths directly linked to plague, which we cannot further refine from the records.

In Fig. 2b we show the time-series of deaths belonging to the *Sant’Eufemia* parish, distinguishing between those caused by the plague and the ones possibly due to other causes. Surprisingly, the first peak of the epidemic begins with few references to the common symptoms of the plague (October to November), when the records point instead to more generic and common illnesses, such as fever or spasms^17^. Only afterwards the records start to extensively mention the plague as the cause of death, well into the Fall of 1631. This might indicate an initial reticence to acknowledge the epidemic outbreak, as well as a subsequent possible overemphasis of it. This reticence might be caused by the public authorities’ practice to quarantine the whole household in their house when someone from it died of plague. It might also be due to a surveillance issue generating a bias in the records: while many deaths were occurring, medical examination was no longer taking place and the registrations of the causes of death were not happening regularly, but instead in batches, leading to approximations. Furthermore, several people were moved to quarantine areas (*lazzaretti*) and died there, while their registration happened subsequently, possibly by reporting generic causes of death. It is thus likely that these deaths are also in large part attributable to plague. However, other explanations are also possible, such as a known epidemic of smallpox co-occurring during the main peak^16^. Despite these limitations and open questions, *Sant’Eufemia*’s causes of death confirm the duration of the epidemic well into the autumn of 1631.

We further verify that deaths by plague were not significantly affected by sex, under the reasonable assumption that sexes were equally distributed in the population of Venice at the time^20^. Indeed, the male to female deaths ratio was close to one ($N_{male} / N_{female} = 865/917 \sim 0.94$), a result confirmed by the majority of the literature^1,12,22–25^, with few exceptions^13,16^. Furthermore, the distribution of illness duration and of age at death did not significantly change with sex (see Figure SI3a and SI3b respectively). Assessing the effect of the plague on age is challenging, as assumptions on the age distribution of population at that time are quite difficult to make and historical statistics are hard to find. Furthermore, the literature on the effect of the plague on different age cohorts is still ambiguous. Nevertheless, our data are in line with previous studies^1,18,26–28^ indicating that the plague had higher relative impact among age cohorts of typically low mortality, in particular adolescents and adults between 14 and 44 years of age, as shown in Figure SI3c and Figure SI3d.

Figure 2c shows the heatmap of reported cases, for each of the parishes of Venice, for the entire time window ($N_{tot\_deaths} = 43088$). One can see that while the main outbreak occurring in the last four months of 1630 shows good synchronicity across all parishes, the second, smaller outbreak occurring until fall 1631 seems to have peaked at rather different time points within each parish, between February and July 1631. We therefore investigate whether space patterns are present, especially in the 1631 outbreaks ($N_{deaths\_tail} = 10363$). In order to assess the presence of spatial patterns, we simply plot the pairwise correlation among cases for all couples of parishes, against the distance between parishes (Figure SI4). The resulting scatter plots show no spatial patterns. Nevertheless, the secondary outbreak in 1631 does not seem to have peaked as homogeneously as the first large outbreak in 1630 (Fig. 2c). We hence performed a clustering analysis to highlight possible groups of rather synchronous parishes (Fig. 3). The analysis on the main 1630 outbreak (Fig. 3a) appears instead to be in sync across parishes.

The clustering on the 1631 epidemic (Fig. 3b) shows outbreaks with more spread-out peaks, across the first half of 1631, with tails reaching the fall of the same year. The main cluster is the one led by the three populous parishes of *S. Geremia, Angelo Rafael* and *S. Nicola*, with peaks between March and May 1631 (central part of Fig. 3b). Another cluster is the one led by the *S. Eufemia* and *S. Marcuola* parishes (bottom part of Fig. 3b), a more heterogeneous group, with peaks occurring mostly in June/July 1631.

**Figure 4:**
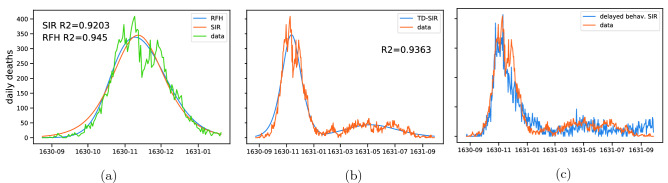
(**a**) Best fit comparison of a simple SIR model against the model from^29^ on the main outbreak peak (150 days time window). (**b**) Best fit of an explicit time-dependent SIR; parameters are shown in Figure SI5d. (**c**) Example realization of a stochastic delayed behavioral SIR; the evolution of transmission rate $\beta (I)$ is shown in Figure SI5e.

Even though these clusters seems to be well separated in time, there is no clear evidence of a specific process or event in the history of the city that might have driven this spatial distribution of localized epidemics in different parishes during 1631. We therefore assess epidemiological models on data aggregated over all parishes. On the other hand, the temporal spreading can be due to few different, non-mutually excluding phenomena, such has the stochasticity of the diffusion itself, as well as the possibility of external introduction, due to the maritime activities of the Republic of Venice. The plague is generally modeled as a zoonosis, in which the transition from an epizootic (typically, in rodents) to a human epidemic is mediated by animal fleas, the vector carrying *Yersinia Pestis*^29,30^. From here on, we refer to this model as the Rats-Fleas-Humans (RFH) model. At the same time, other studies suggest that these models are not always optimal to explain the outbreaks dynamics, especially due to the ‘efficacy and speed’ of some historical plague outbreaks^1^, if compared to the typical dynamics of RFH models. We first confirm that neither a deterministic RFH nor a deterministic Susceptible-Infected-Removed (SIR) model can explain the presence of the 1631 secondary outbreaks (see Figure SI5b). We then investigate the transmission nature of the Venice plague, by considering separately the main 1630 outbreak and the one in 1631. In both cases, we find that the RFH model did not perform much better than a simple SIR model, as shown in Fig. 4a (main 1630 outbreak), and Figure SI5c (1631 outbreaks). We therefore implement a time-dependent SIR and find that it can better explain the dynamics over the entire time window (Fig. 4b), with an increase in the basic reproduction number that could indicate a change in the transmission mechanism of pathogen (for clarity, fitted parameters are reported in Figure SI5d). In particular this might suggest a transition from bubonic to pneumonic plague, a shift already hypothesized for other historical plague epidemics^31^. However, a change in the effective transmission rate might also be due to people’s behavioral response to the outbreak. In order to investigate the fitness of such hypothesis, we implement a stochastic delayed behavioral SIR (details can be found in the Methods). In Fig. 4c we show one example of such model’s stochastic realizations, which presents both a main peak and a long tail dynamics. This shows that a change in pathogen’s transmission route is not necessarily required in order for the epidemic to show a non-trivial temporal pattern, such as the one present in our data. For the sake of completeness we also check whether a deterministic delayed behavioral SIR would fit our data. In Figure SI6b, we show that it cannot actually reproduce the 1631 tail, in spite of a good fit of the first part of the 1630 outbreak.

Although a change in diffusion parameters seems to provide a reasonable explanation of the two-peak structure, we investigate the possibility of having two-peak outbreaks similar to the observed one, as a result of the stochastic nature of the disease spread combined with structural properties of the host network. It is known that the community structure of a network can strongly impact epidemic dynamics^32^. We therefore perform a series of stochastic simulations of a simple SIR process on top of a small-word graph, a network model which is likely to resemble the modular structure of social contacts^33^ (further details on the simulations are given in the Methods). We find that few simulated epidemics do resemble the data, as shown in Figure SI6a. However, as this happens in only about $0.1\%$ of the simulations, such alternative interpretation of the 1631 tail based on pure stochastic effects and network structure, although reasonable, remains very unlikely.

In summary, we find a novel epidemic pattern of two peaks in the 1630–1631 plague outbreak in Venice. The first peak in 1630 was very high, and the outbreak highly synchronized among all parishes; the second peak in 1631 shows temporal variability, and was much less pronounced in strength. Most previous recorded cases show a single main peak^5,6,29^ of varying duration^12,18^, with possible cyclical recurrence^6^. Relying on fine-grained daily death records^1^, we are able to confirm that the plague spanned both the main peak and the long tail, over a period of more than a year and caused the death of approximately 30% of the city’s population.

Providing an interpretation of the two-stage process remains challenging with the evidence at our disposal. Firstly, not all deaths could be clearly attributed to the plague during the early weeks of the main peak. Generic causes of death such as fever and spasms might indicate plague deaths as well as deaths due to other causes. A first hypothesis is therefore that the same plague epidemic went on for more than a year, while being aggravated by other concomitant causes during the main peak. An alternative hypothesis is that two distinct plague epidemics took place instead, one during the main peak and another during the long tail. Previous studies suggest the possibility of a transition from a mainly bubonic to a mainly pneumonic plague, for example. Furthermore, we show that it is also possible that such temporal pattern could be generated by the adaptation of hosts’ behavior to the increase of number of infected, effectively decreasing the transmission rate, as the outbreak advances. Lastly, social factors such as the timing and effectiveness of public containment policies could have played a role.

Further investigations will be needed in order to fully qualify the Venetian 1630–1631 plague outbreak, as well as the Second Pandemic overall^34^. Indeed, as we have shown, historical records contain information which has so far been relied upon only to study few episodes but, when digitized and made available at scale and systematically, can help cast new light on these long-lasting research issues. For an understanding of detailed local dynamics, but also of global patterns of disease spread, modern human data and animal research can now be complemented with digital data collection driven by the digital and medical humanities.

## Methods

### Data collection

The main dataset we consider consists of the daily number of deaths per parish, from January 1629 to December 1631. We have first proceeded with a full double-blind counting, then compared the two series, checking and correcting all discrepancies. Secondly, two different co-authors have counted again all deaths from a sample of 20 parishes out of 70 (8 and 12 each), to further assess our main dataset, with the following results:

- 1629: 22 errors over 2395 assessed registrations (0.91%).
- 1630: 60 over 8989 (0.66%).
- 1631: 16 over 3730 (0.42%).

Confirming that the main dataset was already of high quality. Eventually, all remaining errors were checked again and corrected in the final dataset, which we analyze in this contribution.

We note that the parson of every parish was supposed in principle to (a) get a medical inspection of every dead body to rule out contagious causes, (b) report all deaths every morning to the magistrate called *Provveditori alla Sanità*, (c) get burial licenses from this magistracy before inhumation. Steps (a) and (c) usually were not taking place during the months of peak mortality at the end of the year 1630. It is important to clarify that our death records include deaths which occurred in the main care institutions in Venice: the four *Ospedali Grandi* (main hospitals), as well as minor ones, with respect to residents in the available parishes. They also include all deaths occurred at the *lazzaretti*: temporary locations setup for quarantine or inhumation of persons affected by the plague. They do not include foreigners. We finally note that the parish of *S. Nicola* is to be identified with *San Nicola dei Mendicoli*.

### Data analysis and modeling

All data analysis and modeling are done in Python. For the general data cleaning we use the pandas package. The distance between two parishes is defined as the geodetic distance between the centers of the corresponding polygons, defining the jurisdiction of the same parishes. The geodesic function from the geopy.distance module is used for this task.

All dendrograms (Fig. 3) are plotted using the seaborn.clustermap package. In particular, we use the metric correlation (For more details, find here the description of possible metrics: scipy.spatial.distance.pdist.) and the method complete to build the linkage matrix, needed to compute the clusters.

The compartmental epidemic models are integrated using the odeint function from the scipy.integrate module. The parameters estimations are then obtained using the curve_fit and differential_evolution function from the scipy.optimize package^35^. In order to account for false positives, we estimate a baseline of deaths very likely to be unrelated to the plague outbreak, by fitting a sinusoidal signal from the beginning of the recordings, until the end of August, as shown in Figure SI5a. In the time-dependent SIR model we assumed a simple step function dependence for both $\beta (t)$ and $\gamma (t)$, leading to a total of five fitted parameters: $\beta _1, \beta _2, \gamma _1, \gamma _2$ and the transition time $\tau$ (see again fig SI5d).

Stochastic simulations in Fig. 4 and Figure SI6a were done using the ndlib package^36^, on graphs generated with the networkx package^37^.

The delayed behavioral SIR model (Fig. 4) was defined using the following expression for the transmission rate $\beta (t) = \beta _{0} e^{- I(t - \tau ) / I^{*}}$, where $\beta _{0},\tau$ and $I^{*}$ were fitted parameters, together with the usual (constant) death rate $\gamma$ and initial number of infected $I_{0}$ ($\beta _{0} = 0.06429$, $I^{*} = 72$, $\tau = 32$, $\gamma = 0.02859$, $I_{0} = 3$). For its stochastic implementation we used a Erdos-Renyi graph, with an edge creation probability $p = 4 / N_{nodes}$ ($N_{nodes} = 20{,}000$).

## Supplementary information

Supplementary Information.

## Data Availability

All code and data needed to reproduce plots and analysis presented in the manuscript will be made available in a dedicated GitHub repository (https://github.com/ggrrll/Venice-plague-epidemic-paper).
