# Supplementary material for: A digital reconstruction of the 1630–1631 large plague outbreak in Venice
Source: Sci Rep. 2020 Oct 20;10:17849. doi: 10.1038/s41598-020-74775-6 (PMC7576796; doi:10.1038/s41598-020-74775-6)
Supplement: Supplementary file 1 — Supplementary Information. [file 41598_2020_74775_MOESM1_ESM.pdf]

Supplementary Information
Death in Venice: A Digital Reconstruction of a Large Plague Outbreak During 1630-1631

Gianrocco Lazzari[1,*,+], Giovanni Colavizza[2,*,+], Fabio Bortoluzzi[3], Davide Drago[3], Andrea Erboso[3], Francesca Zugno[3], Frédéric Kaplan[3], Marcel Salathé[1]

[1] Digital Epidemiology Laboratory, School of Life Sciences, École Polytechnique Fédérale de Lausanne (EPFL), Switzerland.
[2] University of Amsterdam, Netherlands.
[3] Digital Humanities Laboratory, College of Humanities, École Polytechnique Fédérale de Lausanne (EPFL), Switzerland.
[*] gianrocco.lazzari@epfl.ch; g.colavizza@uva.nl.
[+] Equal contribution.

"*Sia laudato il signor Iddio non ci sono stati morti.*"
Bless the Lord, there have been no deaths [today].

December 24th 1630, in *Sant'Eufemia*, Venice.

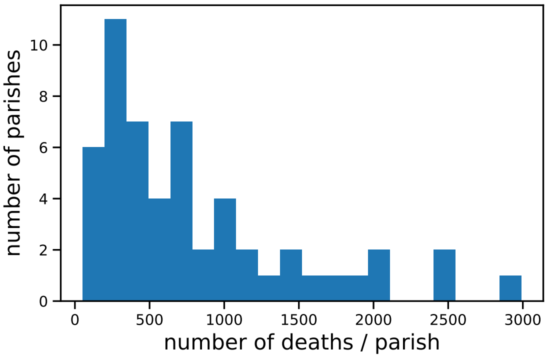

(a)

Parish	N deaths	As fraction of total deaths
S. Eufemia	2089	4.8 %
S. Nicola	2097	4.9 %
S. Geremia	2405	5.6 %
S. Marcuola	2491	5.8 %
S. Pietro di Castello	2990	6.9 %

(b)

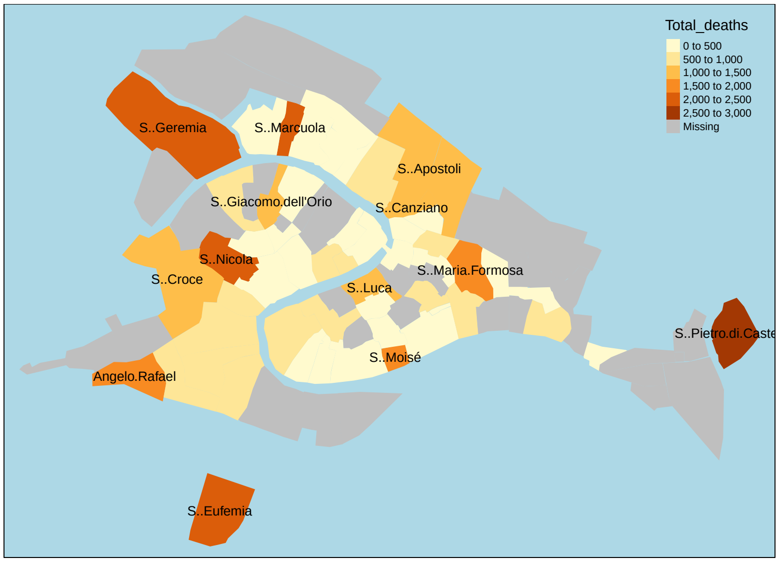

(c)

Supplementary Figure SI1: Distribution of number of deaths by parish. (a) One can clearly see the skewed distribution, with the top 24% of parishes accounting for about 55% of the total deaths. (b) For the sake of clarity, only parishes with more than 2000 deaths are listed. (c) Map of Venice parishes, color-coded by total recorded deaths, summed over the entire time-window. For clarity, only the names of parishes with more than 1000 total deaths are shown.

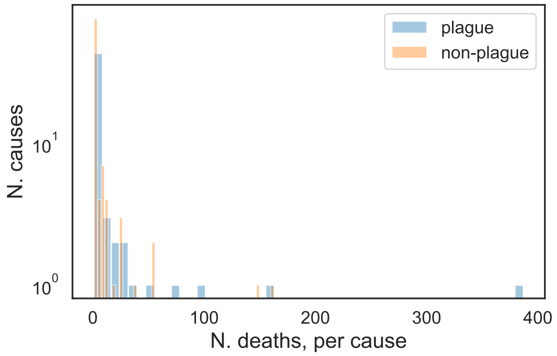

(a)

Cause of death	N deaths	As fraction
**mal sospetto**	387	21.7 %
febbre	163	9.1 %
**petecchie nere**	157	8.8 %
spasimo	148	8.3 %
**contagio**	100	5.6 %
**mal contagioso**	73	4.1 %
vermi	56	3.1 %
nascente	54	3.0 %

(b)

Supplementary Figure SI2: Distribution of number of deaths by cause, for the parish of *Sant'Eufemia*. In the records, 156 unique causes of death are found, of which 56 were attributed to plague. The distribution of attributed causes is skewed
, with the top 5% of causes accounting for about 63% of the overall deaths (a). For the sake of clarity, only causes with more than 50 deaths are listed; in bold the ones attributed to plague (b).

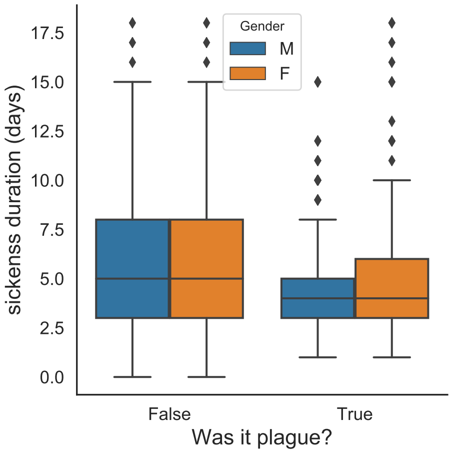

(a)

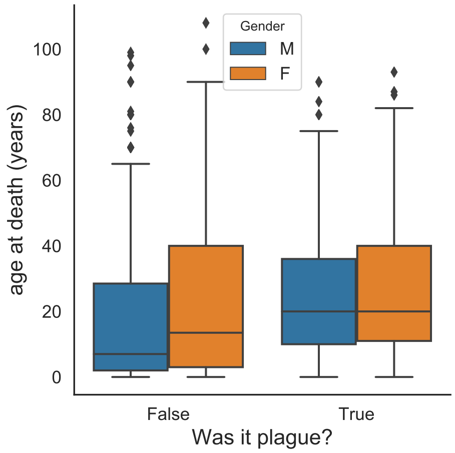

(b)

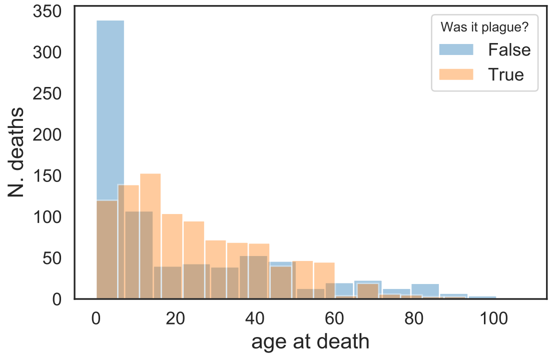

(c)

Age cohort (years old)	Plague	Not plague	Aggregated
Infant (0-2)	3.38%	23.52%	12.16%
Child (3-13)	32.01%	29.2%	30.42%
Young (14-23)	20.95%	8.74%	15.63%
Adult (24-44)	29.2%	16.45%	23.64%
Old (45+)	17.28%	19.28%	18.15%

(d)

Supplementary Figure SI3: Demographic statistics for the parish of *Sant'Eufemia*, from the 1630-31 records. (a) Distribution of sickness duration, by sex and cause of death for *S. Eufemia* death records – for sake of clarity the boxplots include only cases with a sickness spanning less than 20 days (this still covers about 88% of the total sickness duration distribution). (b,c) Distributions of age at death, divided by cause of death. No significant age difference emerges due to sex ($p > 0.001$ on two samples KS test, for both causes of death) (b), while a significant one appears between the plague VS non-plague deaths, aggregated over sex ($p < 10^{-20}$ on two samples KS test) (c). (d) Table with the same numbers of deaths, divided into age groups. Note that an infant mortality at birth between 20 and 30% was common at the time [1].

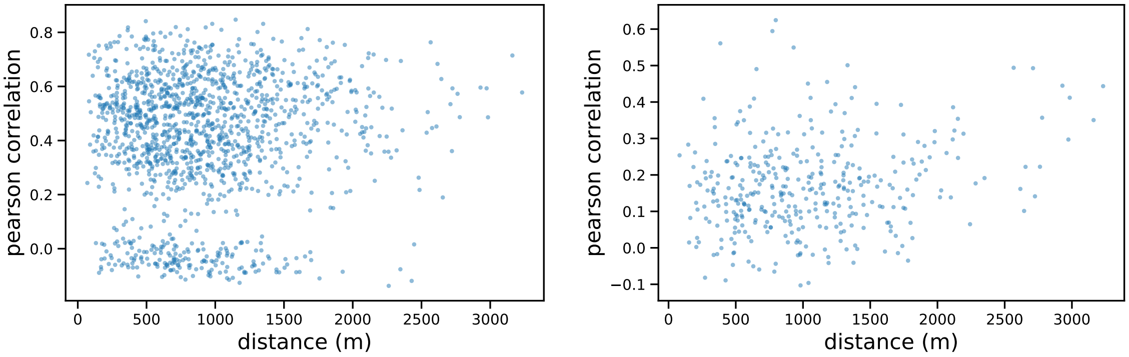

(a) (b)

Supplementary Figure SI4: Pairwise Pearson correlation between cases time-series of each couple of parishes (y-axis) against the distance between the two parishes (x-axis). The same scatter plot for all parishes ($N_{parishes} = 54$) for the entire time-windows (a) and for the largest parishes, for the 1631 outbreaks only (b). The largest parishes are defined as those reporting more than 500 deaths ($N_{parishes} = 28$).

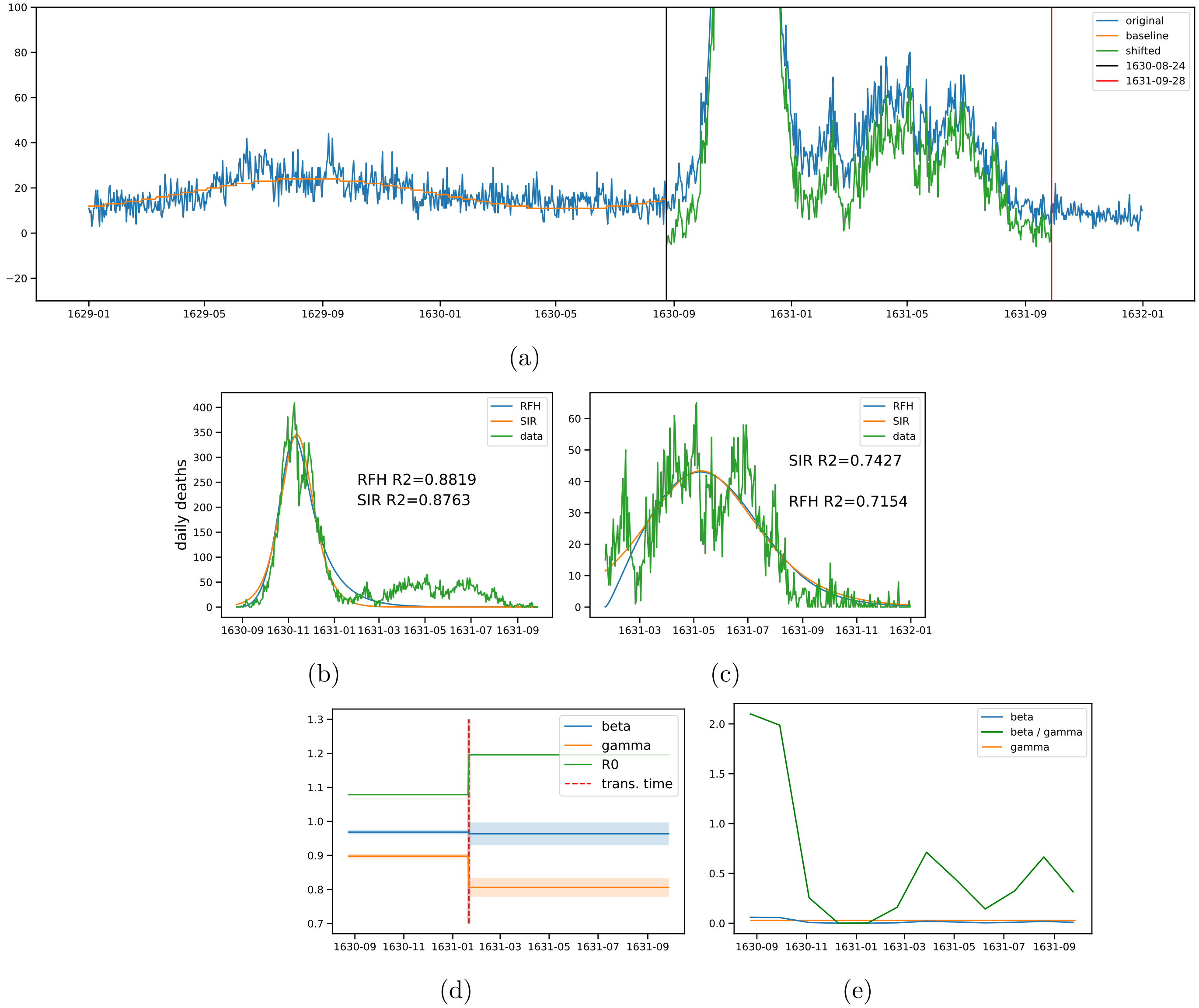

Supplementary Figure SI5: (a) Estimation of baseline cases due to other causes: a sinus function (orange line) is fitted from the beginning of the data until the beginning of the fit (black vertical line) to estimate the mortality rate, which is then applied to the original data (blue) to get the data (green line) used for the fit (red vertical line indicates the estimated end of the epidemic). (b-c) Comparison between a simple SIR and the more complex RFH model, in the 400 days window (b) and zoomed on the second part of the epidemic (c). (d) Fitted parameters for the time-dependent SIR model, as in Figure 4b. (e) Evolution of fitted $\beta(I)$ and $\beta(I)/\gamma$ for the delayed behavioral SIR shown in Figure 4c.

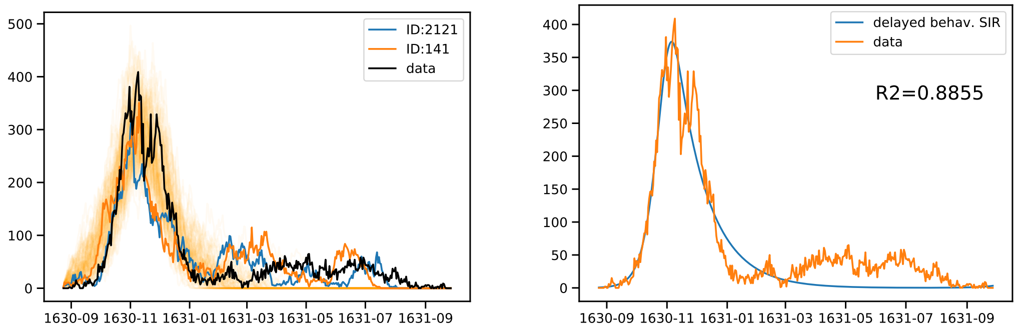

(a)

(b)

Supplementary Figure SI6: (a) Selected stochastic simulations of a simple SIR model on top of a small-word network. Two particular epidemics are highlighted, in order to show the possibility of having a large peak followed by a long tail, as present in the data. In shaded orange we only show, for sake of clarity, the simulated epidemics with lowest deviation from the data ($RMSE < 50 - N = 93$) (b) Best fit of deterministic behavioral delayed SIR. Although the model can fit very well the first part of the epidemic, it does not show a secondary outbreak, in 1631.

**Death causes**	**Related to plague ?**
annegato	False
apoplessia	False
brusco	False
bubbone pestilenziale all'inguine	**True**
caduta	False
caduta	False
caduta apoplettica	False
caduto da una scala	False
caita nella gola	False
cancro alla bocca	False
carbone	**True**
carboni	**True**
carboni e parto	**True**
carbonie e petecchie nere	**True**
catare nella gamba	False
catarro	False
contagio	**True**
contagio e petecchie nere	**True**
convertito etico	False
croplesion	False
disperso	False
doglia di testa	False
doglia di testa e mazzucco	**True**
doglia di testa e vermi	False
doglia e spasimo	False
dolor di vita	False
febbre	False
febbre	False
febbre continua	False
febbre continua e altre indisposizioni	False
febbre e catarro	False
febbre e doglia	False
febbre e doglia di schiena	False
febbre e doglie di testa	False
febbre e ferita	False
febbre e flusso	False
febbre e gotta	False
febbre e lepra(?)	False
febbre e mazzucco	**True**
febbre e nosella	False
febbre e petecchie	**True**
febbre e petecchie nere	**True**
febbre e petecchie rosse	**True**
febbre e petecchie rosse non pestilenziali	False
febbre e ponta	False
febbre e spasimo	False
febbre e suspetto	**True**
febbre e un brusco	False
febbre e una doglia in un fianco	False
febbre e una postiema	False
febbre e una scorencia	False
febbre e variole	False
febbre e vecchiezza	False
febbre e vermi	False
febbre etica	False
febbre etica e catarro	False
febbre ferita e flusso	False
febbre galica e catarro	False
febbre maligna	**True**
febbre maligna e mal sospetto	**True**
febbre maligna e punti	**True**
febbre senza sospetto	False
ferita	False
ferita	False
ferita dietro l'orecchio	False
ferite	False
ferite da peste	**True**

Supplementary Table SI1: Manual classification of all 156 death causes as reported in necrologies, as associated to plague or not – part 1.

Death causes	Related to plague ?
ferito	False
flusso	False
fracassato la testa	False
illegibile	False
incinta da febbre e doglie	False
infermo	False
ipolesia	False
ipoplessia e febbre	False
macchie nel petto giudicate pestilenziali	**True**
mal caduco	False
mal caduco e vermi	False
mal contagioso	**True**
mal di febbre	False
mal di gotta	False
mal di mare e mal sospetto	**True**
mal di mazzucco	**True**
mal di pietra	False
mal di reni	False
mal mazzucco	**True**
mal sospetto	**True**
mal sospetto e petecchie	**True**
mazzucco	**True**
morto improvvisamente	False
n.d.	False
nascente	False
non aver latte	False
non si sa il male	False
nosella	False
nosella di mal contagioso	**True**
nosella nel cuore	False
paralitico senza contagio	False
parto	False

Death causes	Related to plague ?
parto e febbre	False
parto e ferita	False
parto e spasimo	False
partorito morto	False
patimento	False
per non aver avuto latte	False
percossia	False
peste	**True**
peste e petecchie nere	**True**
peste e strupiata	**True**
petecche paonazze	**True**
petecchi nere	**True**
petecchie	**True**
petecchie	**True**
petecchie e febbre maligna	**True**
petecchie e mazzucco	**True**
petecchie e spasimo	**True**
petecchie e un brusco	**True**
petecchie et un brusco	**True**
petecchie nere	**True**
petecchie nere	**True**
petecchie nere contagiose	**True**
petecchie nere e carbone	**True**
petecchie nere e rosse	**True**
petecchie nere pestilenziali	**True**
petecchie pestilenziali	**True**
petecchie rosse	**True**
petecchie rosse e alcune nere	**True**
petecchie rosse e parto	**True**
petecchie rosse verso il nero	**True**

Supplementary Table SI2: Manual classification of all 156 death causes as reported in necrologies, as associated to plague or not – part 2.

**Death causes**	**Related to plague ?**
punta	False
rogna	False
sconosciuta	False
sempre infermo	False
senza peste esterna	False
senza sospetto	False
spasimo	False
spasimo e infermitá	False
spasimo e mazzucco	**True**
spasimo e petecchie nere	**True**
spasimo e sturioli	False
spasimo e vecchiezza	False
spasimo e vermi	False
stroppiata su la palada	False
strupiata	False
tumore	False
tumore alla gola	False
un carbon	**True**
variole	False
variole e sturioli	False
vecchiezza	False
vecchiezza e febbre	False
vermi	False
vermi e petecchie	**True**
vermi e spasimo	False

Supplementary Table SI3: Manual classification of all 156 death causes as reported in necrologies, as associated to plague or not – part 3.

References

[1] Weiner Gordon M. The Demographic Effects of the Venetian Plagues of 1575-77 and 1630-31. *Genus*, 26(1/2):41–57, 1970.
